# Down-Regulation of Long Non-Coding RNA TINCR Induces Cell Dedifferentiation and Predicts Progression in Oral Squamous Cell Carcinoma

**DOI:** 10.3389/fonc.2020.624752

**Published:** 2021-02-25

**Authors:** Zehang Zhuang, Jing Huang, Weiwang Wang, Cheng Wang, Pei Yu, Jing Hu, Haichao Liu, Hanqi Yin, Jinsong Hou, Xiqiang Liu

**Affiliations:** ^1^Department of Oral and Maxillofacial Surgery, Guanghua School of Stomatology, Hospital of Stomatology, Sun Yat-sen University, Guangzhou, China; ^2^Guangdong Provincial Key Laboratory of Stomatology, Sun Yat-sen University, Guangzhou, China; ^3^Department of Oral and Maxillofacial Surgery, Xiangya Hospital, Central South University, Changsha, China; ^4^Department of Prothodontics, Affiliated Stomatology Hospital of Guangzhou Medical University, Guangzhou, China; ^5^Guangzhou Key Laboratory of Basic and Applied Research of Oral Regenerative Medicine, Guangzhou, China; ^6^State Key Laboratory of Biocontrol, School of Life Sciences, Sun Yat-Sen University, Guangzhou, China; ^7^South China Institute of Biomedine, Guangzhou, China; ^8^Department of Oral and Maxillofacial Surgery, NanFang Hospital, Southern Medical University, Guangzhou, China

**Keywords:** lncRNAs (long non-coding RNAs), TINCR (tissue differentiation-inducing non-protein coding RNA), OSCC (oral squamous cell carcinoma), cell differentiation, metastasis, progression

## Abstract

**Objectives:**

Recently long non-coding RNAs (lncRNAs) have emerged as novel gene regulators involved in tumorigenic processes, including oral squamous cell carcinoma (OSCC). Here, we identified a differentiation-related lncRNA, terminal differentiation-induced non-coding RNA (TINCR). However, its biological function and clinicopathological significance in OSCC still remain unclear.

**Methods:**

The lncRNA expression profiles in OSCC tissues and paired adjacent non-tumor tissues (NATs) from 10 patients were detected by lncRNA microarrays. Weighted gene co-expression network analysis (WGCNA) and gene ontology (GO) enrichment were performed to identify the most significant module and module functional annotation, respectively. Potential differentiation-related lncRNAs were screened by differential expression analysis. TINCR was further confirmed in OSCC cell lines and tissues of another patient cohort by using qRT-PCR. The correlation between the TINCR expression level and clinicopathological characteristics was analyzed. The effects of TINCR on cell differentiation, migration and invasion were assessed by knockdown or knock-in *in vitro* and *in vivo*.

**Results:**

WGCNA and GO enrichment analysis showed that one co-expression network was significantly enriched for epithelial cell differentiation, among which, TINCR was significantly downregulated. qRT-PCR analyses validated down-regulation of TINCR in tumor tissues compared with paired NATs, and its expression was closely correlated with pathological differentiation and lymph node metastasis in patients with OSCC. Patients with lower TINCR expression levels had worse survival. Cell function experiments showed that TINCR played a crucial role in epithelial differentiation. Both TINCR and epithelial differentiation-associated genes, including IVL and KRT4, were significantly upregulated during OSCC cell calcium-induced differentiation but were reduced when cell dedifferentiation occurred in tumor spheres. Overexpression of TINCR dramatically suppressed cell dedifferentiation, migration and invasion *in vitro*, while knockdown of TINCR had the opposite effects. Upregulation of TINCR significantly elevated the expression of terminal differentiation genes and repressed tumor growth *in vivo*. Moreover, TINCR significantly suppressed the activation of JAK2/STAT3 signaling in OSCC cells.

**Conclusion:**

Our study suggests that TINCR functions as a tumor suppressor by inducing cell differentiation through modulating JAK2/STAT3 signaling in OSCC. TINCR may serve as a prognostic biomarker and therapeutic target for OSCC.

## Introduction

Head and neck cancer is the sixth most common malignancy and is a major cause of cancer morbidity and mortality worldwide (1). Oral squamous cell carcinoma (OSCC), comprising a major portion of head and neck cancers, accounts for approximately 90% of all oral malignancies (2). Based on the statistics from the American Cancer Society (http://seer.cancer.gov/statfacts/html/oralcav.html), an estimated 48,000 new OSCC cases occurred in 2016, composing 3% of all new malignancies (3). Despite aggressive treatment methods, including radiation therapy, chemotherapy and surgery, the 5-year survival rate remains at 50–55%, with little improvement due to rapid metastasis and a high regional relapse rate (4). Thus, there is an urgent need for a deeper understanding of OSCC pathogenesis to develop effective therapeutic approaches.

Long non-coding RNAs (lncRNAs) were first described during the large-scale sequencing of full-length cDNA libraries in the mouse (5) and refer to RNAs with no protein coding potential that are transcribed to RNA molecules longer than 200 nucleotides (6). Although lncRNAs have been extensively studied over the past several decades, very little is known about the specific role of lncRNAs. However, the recent explosion of knowledge highlighting the importance of lncRNAs in the regulation of diverse major biological processes, including but not limited to development, differentiation, and metabolism, has brought these ignored molecular players to the forefront (6).

Terminal differentiation-induced non-coding RNA (TINCR) is an important regulatory molecule for epithelial differentiation. Studies have shown that TINCR regulates the differentiation-related gene KRT80 by forming a TINCR-STAU1 complex (7). Accumulating evidence suggests that TINCR is involved in malignant progression, including tumor growth, metastasis, and chemoradiation resistance (8–12). However, the relationship between TINCR and tumor differentiation and the potential mechanism remain unclear.

In this study, we aimed to explore the role of TINCR in OSCC, especially the relationship between TINCR and tumor cell differentiation. We hoped to find a potential biomarker for predicting the prognosis of OSCC patients that is beneficial for OSCC treatment.

## Materials and Methods

### Patient Tissue Samples

A total of 42 human OSCC specimens were enrolled in this study that were pathologically diagnosed at the Hospital of Stomatology, Sun Yat-Sen University between 2013 and 2015. All patients received radical surgery without receiving any form of pre-surgical adjuvant therapy. All patients were given written informed consent for the purposes of the study. The study was approved by the Ethical Committee of Hospital of Stomatology, Sun Yat-sen University. All studies of patient specimens were conducted in accordance with the Declaration of Helsinki.

### Cell Lines

The human OSCC cell lines SCC9, SCC15, and CAL27 were obtained from ATCC (Rockville, MD, USA). UM1, UM2 and SCC1 were provided by Dr. Xiaofeng Zhou (University of Illinois at Chicago, IL, USA). HSC3, HSC6, HN6, CAL33 and normal oral keratinocytes (NOK) were kindly provided by J. Silvio Gutkind (NIH, Bethesda, MD, USA). UM1, UM2, SCC9, SCC15 and SCC25 cell lines were cultured in Dulbecco’s modified Eagle’s medium/Ham’s F12 (Gibco, Rockville, MD, USA) supplemented with 10% fetal bovine serum (FBS, Gibco). SCC1, HSC3, HSC6, HN6, CAL27 and CAL33 cells were cultivated in Dulbecco’s modified Eagle’s medium (DMEM, Gibco) supplemented with 10% FBS (Gibco). NOK cells were grown in keratinocyte serum-free medium containing human recombinant epidermal growth factor and bovine pituitary extract (Life Technologies). All cells were incubated at 37°C in a humidified atmosphere with 5% CO_2_. All cell lines were routinely tested for Mycoplasma with PlasmoTest^TM^ Mycoplasma contamination detection kit (InvivoGen).

### mRNA and lncRNA Microarray

Total RNA was amplified and labeled with a Low Input Quick Amp Labeling Kit, One-Color (Agilent Technologies, Santa Clara, CA, USA) following the manufacturer’s instructions. Labeled cRNA was purified with an RNeasy mini kit (QIAGEN, Duesseldorf, Germany). Each slide was hybridized with 1.65 μg Cy3-labeled cRNA using a Gene Expression Hybridization Kit (Agilent Technologies) in a hybridization oven (Agilent Technologies) according to the manufacturer’s instructions. After 17 h of hybridization, slides were washed in staining dishes (Thermo Shandon, Waltham, MA, USA) with a Gene Expression Wash Buffer Kit (Agilent Technologies). Slides were then scanned by an Agilent Microarray Scanner (Agilent Technologies) with default settings (dye channel: green, scan resolution = 3 μm, 20 bit). Data were extracted with Feature Extraction software 10.7 (Agilent Technologies). Raw data were normalized by the quantile algorithm, Gene Spring Software 11.0 (Agilent Technologies).

### Subcellular Fractionation Location

Nuclear and cytoplasmic components were isolated by using the PARIS Kit (Life Technologies, USA) according to the manufacturer’s instructions.

### Quantitative Real-Time PCR (qRT-PCR)

Briefly, TRIzol reagent (Invitrogen, CA, USA) was used to isolate total RNA according to the manufacturer’s instructions. PrimeScript^TM^ RT Master Mix (TaKaRa, Japan) was used to perform reverse transcription. qRT-PCR was performed using SYBR GREEN I Master Mix (Roche, Basel, Switzerland) on a Light Cycler 480 system (Roche) according to the manufacturer’s instructions. After normalization to the GAPDH expression levels, the relative expression levels were calculated using the 2^-ΔΔT^ method. The forward and reverse primer sequences used for qRT-PCR are shown in **Supplementary Table 1**.

### Western Blotting

Western blotting was performed as described previously (13). Specifically, antibodies against KRT4 (Abcam, Cambridge, UK) and IVL (Sigma-Aldrich, St Louis, MO, USA) were used as primary antibodies at a dilution of 1:1000. GAPDH (1:1000, Cell Signaling Technology, Danvers, MA, USA) was used as an internal reference. The target proteins were detected with an ECL kit (Millipore, Billerica, MA, USA) and quantified with ImageJ software analysis.

### RNAScope Assay

The RNAScope targeting probe of TINCR was designed and synthesized by Advanced Cell Diagnostics, and the analysis of TINCR expression was performed with the RNAscope^®^ 2.5 High Definition (HD) Detection Reagent-BROWN (Advanced Cell Diagnostics, USA) according to the manufacturer’s instructions. The images were acquired by an Aperio ImageScope (Leica Biosystems, Germany).

### Cell Transfection

Transient transfection was performed using Lipofectamine RNA iMAX Transfection Reagent (Invitrogen) according to the manufacturer’s instructions. TINCR siRNA was designed and synthesized by GenePharma (Shanghai, China). The sequences were designed as follows: sense, 5’-GGUACUGGCUGAAGGAAUATT-3’; antisense, 5’-UAUUCCUUCAGCCAGUACCTT-3’. The TINCR lentiviral vector (pEZ-TINCR) and control vector were constructed by GeneCopoeia (Rockville, MD, USA).

### Transwell Assay

Cell migration and invasion were measured with a BD BioCoat^TM^ system (BD Biosciences, San Jose, CA, USA). The assays were performed as previously described (14). Briefly, for migration assays, cells were seeded into the upper chambers without Matrigel-coated membranes and cultured with serum-free medium, while the lower chambers were filled with complete medium. For invasion assays, cells were seeded into the upper chambers with Matrigel-coated membranes. The cells were incubated at 37°C for 24 h. The cells on the top surface of the chamber were removed with a cotton swab, and then the cells on the lower surface of the membrane were fixed in 4% paraformaldehyde for 25 min. Cells were stained with 0.1% crystal violet (Sigma-Aldrich), and the cells were counted under a Zeiss microscope (Germany).

### Wound Healing Assay

Cells were plated into 6-well plates and incubated at 37°C for 24 h. A 200-µL pipette tip was used to make a straight scratch, and the cells was washed with PBS. Then, medium without FBS was added. Images were taken under an inverted microscope (Carl Zeiss).

### Cell Counting Kit-8 Assay

Cell proliferation was analyzed using the Cell Counting Kit-8 (CCK-8, Sigma-Aldrich). Briefly, 2 × 10^3^ cells were seeded in triplicate into a 96-well plate. Cell viability was assessed at 0, 24, 48, 72, and 96 h. The absorbance was measured at 450 nm using a microplate reader (Genios TECAN, Männedorf, Schweiz).

### Tumor Sphere-forming Assay

Tumor sphere-forming assays were performed as previously described (14). In brief, cells were plated into ultra-low attachment 6-well plates with serum-free medium supplemented with 2% B27 (Invitrogen), 10 ng/mL epidermal growth factor (EGF, Invitrogen), and 10 ng/mL basic fibroblast growth factor (bFGF, Invitrogen). The medium was changed every three days until the diameters of spheres exceeded 40 μm.

### *In Vivo* Experiments

All animal studies were conducted with the approval of the Sun Yat-sen University Institutional Animal Care and Use Committee and were performed in accordance with established guidelines. Female BALB/c nude mice aged 4–6 weeks were purchased from the Animal Care Unit of Guangdong and maintained in specific pathogen-free (SPF) conditions. After being suspended in 100 µL sterilized PBS, 1 × 10^6^ HSC3 cells were subcutaneously injected into the armpit of the forelimb. Tumor growth was observed on a regular basis, and the volume was measured with a Vernier caliper. Tumor volume was calculated with the following formula: tumor volume = (length × width × width/2). At 18 days after injection, the mice were sacrificed, and the tumor xenografts were weighed and collected.

### Weighted Gene Co-Expression Network Construction

The expression profile of the microarray was used to construct a gene co-expression network by using the R package “WGCNA”. Pearson’s correlation analysis of all pairs of genes was used to construct an adjacency matrix, which was used to construct a scale-free co-expression network based on the soft-thresholding parameter β. Then, the adjacency matrix was turned into a topological overlap matrix (TOM), which represented the overlap in the shared neighbors to further identify functional modules in the co-expression network.

### Identification of Clinical Significant Modules

The correlation between modules and clinical features was evaluated by Pearson’s correlation coefficient (PCC) analysis. Clinical information included tissue (cancerous or not) and lymph node status (metastatic or not). The correlation between the module eigengenes (MEs) and the clinical features was assessed to identify clinical significant modules.

### Gene Ontology (GO) Enrichment Analysis

GO enrichment analysis was performed on key modules using the R package clusterProfiler. *P*<0.01 was defined as a significant enrichment analysis result.

### Statistical Analysis

All statistical analyses were performed using SPSS 20.0 software (SPSS Inc., Chicago, IL, USA) or GraphPad Prism 8 (La Jolla, CA, USA). Student’s *t*-test, the Wilcoxon test or the *χ*^2^ test was used to analyze two-group comparisons. The Kaplan–Meier method and log-rank test were performed to evaluate survival outcomes. Differences were considered statistically significant at *P* < 0.05.

## Results

### WGCNA Construction and Gene Module Recognition

To screen lncRNAs that are deregulated in OSCC, we comparatively analyzed mRNA and lncRNA profiles of 10 OSCC patient samples and their paired non-cancerous adjacent counterparts. The microarray data were subjected to differential expression analysis. According to the microarray data, we identified 1603 transcripts that were upregulated with a more than 2-fold change (FC) in OSCC samples compared to non-cancerous adjacent tissues (NATs), while 989 transcripts were downregulated by more than 2-fold (**Supplementary Figure 1**).

**Figure 1:**
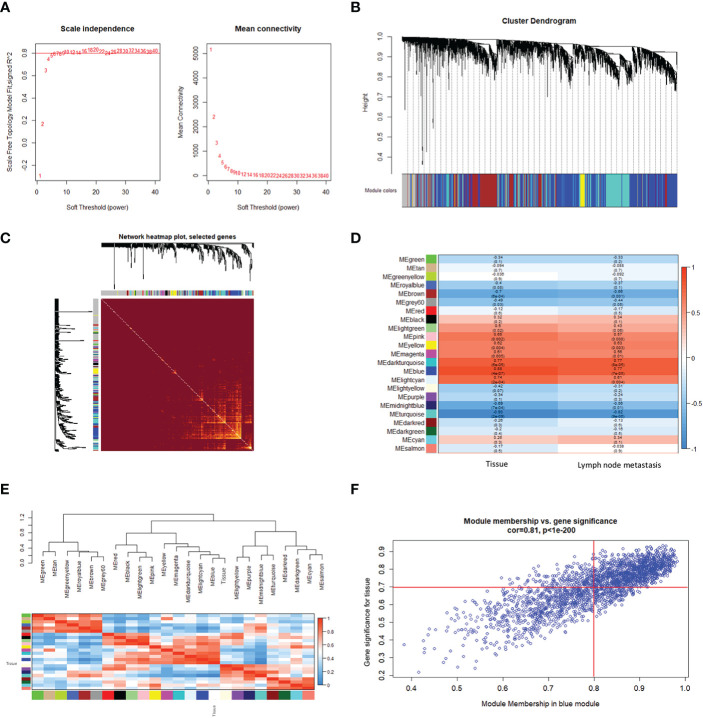
Construction of a weighted gene co-expression network. **(A)** Analysis of the scale-free fitting index for soft threshold powers (β) and the mean connectivity for soft threshold powers. **(B)** Hierarchical clustering dendrograms of identified co-expressed genes in modules in OSCC. Each colored row represents a color-coded module that contains a group of highly connected genes. The gray module indicates none co-expression between the genes. **(C)** Twenty-three significant co-expression gene modules were identified with a topological overlap matrix (TOM) plot. The different colors of the horizontal and vertical axes represent different modules. The yellow brightness in the middle indicates the degree of connection between the different modules. **(D)** Heatmap of the Pearson correlation coefficient (PCC) between module eigengenes (MEs) and clinical information of OSCC. Each cell contains the correlation coefficient and *P*-value. The color of the cell reflects the size of the correlation coefficient, as shown in the legend on the right. **(E)** Correlated heatmap plot of the adjacency modules in the WGCNA. The rectangle of each row and column represents an ME. In the correlated heatmap plot, light blue represents low adjacency, while red represents high adjacency. **(F)** Scatter plot of module eigengenes related to the tissue in the blue module.

To further explore the co-expression patterns of the lncRNAs and mRNAs in OSCC, weighed gene co-expression network analysis (WGCNA) was performed. A total of 16130 genes, consisting of 4549 lncRNAs and 11581 mRNAs, were used for cluster analysis with the WGCNA package. In this study, a power of β = 20 (scale-free R^2^ = 0.80) was selected for the soft-thresholding to ensure the network was scale-free, and 23 modules were obtained for subsequent analysis (**Figure 1A**). Each of the modules was marked by a color, while the gray module was a gene that was not co-expressed (**Figure 1B**).

We constructed a topological overlap matrix (TOM) plot to analyze the interaction among the 23 modules, which demonstrated the relative independence of the modules. We also found the strong co-expression relationships among the genes in the blue module (**Figure 1C**). To determine whether any of the identified expression modules were associated with the progression of OSCC, we explored the relationships between the modules and tissue (cancerous or not), and lymph node status (metastatic or not). As shown in **Figure 1D**, compared with other modules, the blue module, as well as the dark turquoise and turquoise modules, were strongly correlated with the tissue and lymph node status, respectively. Among them, the blue and dark turquoise modules were both positively correlated with the tissue (r = 0.88, *p* = 4e-07 for blue, r = 0.77, *p* = 6e-05 for dark turquoise) and lymph node status (r = 0.77, *p* = 7e-05 for blue, r = 0.77, *p* = 8e-05 for dark turquoise), whereas the turquoise module had the highest negative correlation with the tissue (r = -0.93, *p* = 2e-09) and lymph node status (r = -0.82, *p* = 9e-06) in OSCC. In addition, the heatmap based on adjacencies showed that the blue module was most closely related to the tissue status (**Figure 1E**). The scatter plot also illustrated the associations between the blue modules and the genetic significance (**Figure 1F**). All the results suggest that the blue module is the module that is the most relevant to OSCC progression. Therefore, the blue module was chosen for further analysis.

### Module Functional Annotation and Screening Cell Differentiation Related LncRNAs in OSCC

**Figure 2:**
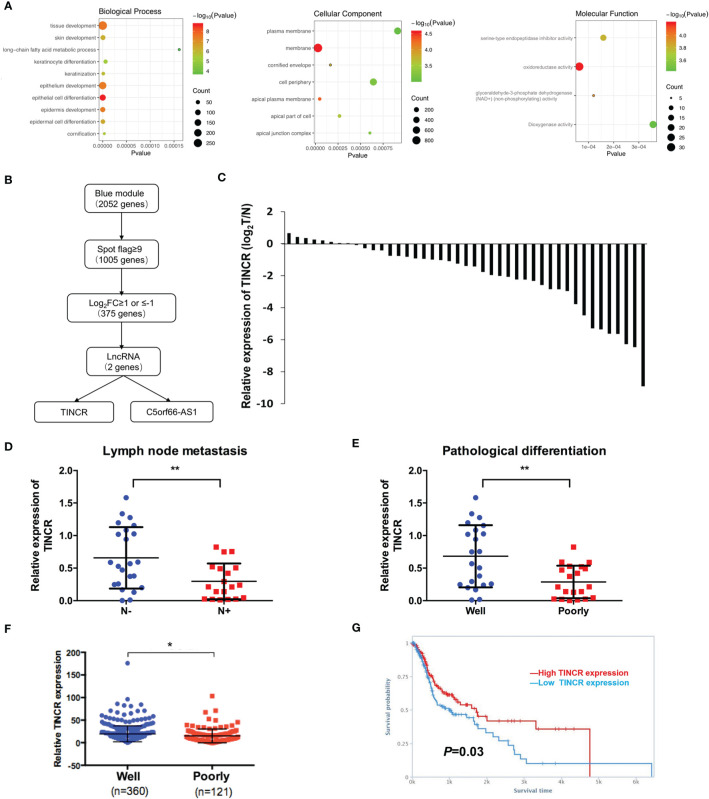
TINCR was downregulated in OSCC tissues. **(A)** Enrichment of the gene ontology (GO) terms of mRNAs in the blue module. **(B)** A workflow of lncRNA screening. **(C)** The expression of TINCR in OSCC tissues and non-cancerous adjacent tissues (NATs) was shown by qRT-PCR. qRT-qPCR analysis of TINCR expression in OSCC samples grouped by the lymph node metastasis **(D)** and the pathological differentiation **(E)**. **(F)** Analysis of TINCR expression in HNSCC samples from the TCGA database, and the HNSCC samples were grouped by the pathological differentiation. **(G)** Kaplan-Meier analysis from TANRIC database based on the HNSCC patient cohort of TCGA showed that the high TINCR expression group had a higher survival rate than the low TINCR expression group. *P < 0.05, **P < 0.01.

To elucidate the biological functions of the module genes, GO enrichment analysis of the blue module was performed by using the “clusterProfiler” R package (**Figure 2A**). The results showed that the principal biological functions of the blue module were closely related to epithelial development and epithelial cell differentiation, which demonstrated that the genes of the blue module were mainly involved in the regulation of epithelial cell differentiation (**Figure 2A**).

Of the 2052 mapped genes in the blue module, 1005 genes with high fluorescence intensity on the Agilent Microarray Platform were identified based on the threshold of spot flag≥9 in 10 OSCC samples or in 10 non-cancerous adjacent counterparts. Then, the 1005 genes were subjected to differential expression analysis (**Figure 2B**). A total of 375 differentially expressed genes (DEGs) were screened under the threshold of false discovery rate (FDR) <0.05 and log_2_FC ≥1 or ≤-1, of which 2 lncRNAs were identified, TINCR and C5orf66-AS1 (**Figure 2B**). Recent studies have revealed that TINCR plays a pivotal role in normal epidermal differentiation. In addition, emerging evidence has suggested that the aberrant expression of TINCR is associated with cell growth, metastasis, and drug resistance in human cancers. However, the specific role of TINCR in the cell differentiation of OSCC is still not fully understood. Therefore, TINCR was selected for further investigation.

### TINCR Was Downregulated in OSCC Tissues and Associated With a Poor Prognosis

To explore the expression of TINCR in OSCC tissues, 42 OSCC samples and their paired non-cancerous adjacent counterparts were employed to detect the expression of TINCR by qRT-PCR. Our results showed that TINCR was dramatically downregulated in OSCC tissues compared to NATs (**Figure 2C**). To further clarify the association of TINCR expression with clinicopathological characteristics in OSCC patients, the 42 patients were divided into a low-expression group (n = 24, 57.1%) and a high-expression group (n = 18, 42.9%) based on the median expression level of TINCR. As presented in **Table 1**, the expression level of TINCR was negatively associated with lymph node metastasis (**Figure 2D**) and positively correlated with pathological differentiation (**Figure 2E**) in OSCC patients. However, no significant association between TINCR expression level and patient sex, age, tumor size, T classification or clinical stage was observed (**Table 1**). To further confirm these findings, we downloaded the RNA sequencing dataset of 481 head and neck squamous cell carcinoma (HNSCC) tissues from the TCGA database. As expected, the expression level of TINCR was significantly lower in poorly differentiated tissues than in well-differentiated tissues (**Figure 2F**). Furthermore, Kaplan-Meier analysis showed that overall survival was significantly worse in the TINCR low-expression group than that in the TINCR high group (**Figure 2G**). These results indicate that down-regulation of TINCR is associated with tumor progression and worse prognosis in patients with OSCC.

**Table 1:** Correlation of TINCR expression with clinicopathological features of OSCC patients (n = 42).

Characteristics	Number	TINCR Expression		*P*
		Low	High	
**Gender**				
Male	26	16	10	0.463
Female	16	8	8	
**Age**				
≤55y	25	15	10	0.650
>55y	17	9	8	
**Tumor size**				
≤4cm	22	12	10	0.721
>4cm	20	12	8	
**LN metastasis**				
Negative	23	10	13	**0.049**
Positive	19	14	5	
**Pathological differentiation**				
Well	22	9	13	**0.026**
Moderately/Poorly	20	15	5	
**T classification**				
T1-T1	22	12	10	0.721
T3-T4	20	12	8	
**Clinical stage**				
I-II	15	8	7	0.710
III-IV	27	16	11	

Bold values means P < 0.05.

### TINCR Was Involved in the Cell Differentiation of OSCC

**Figure 3:**
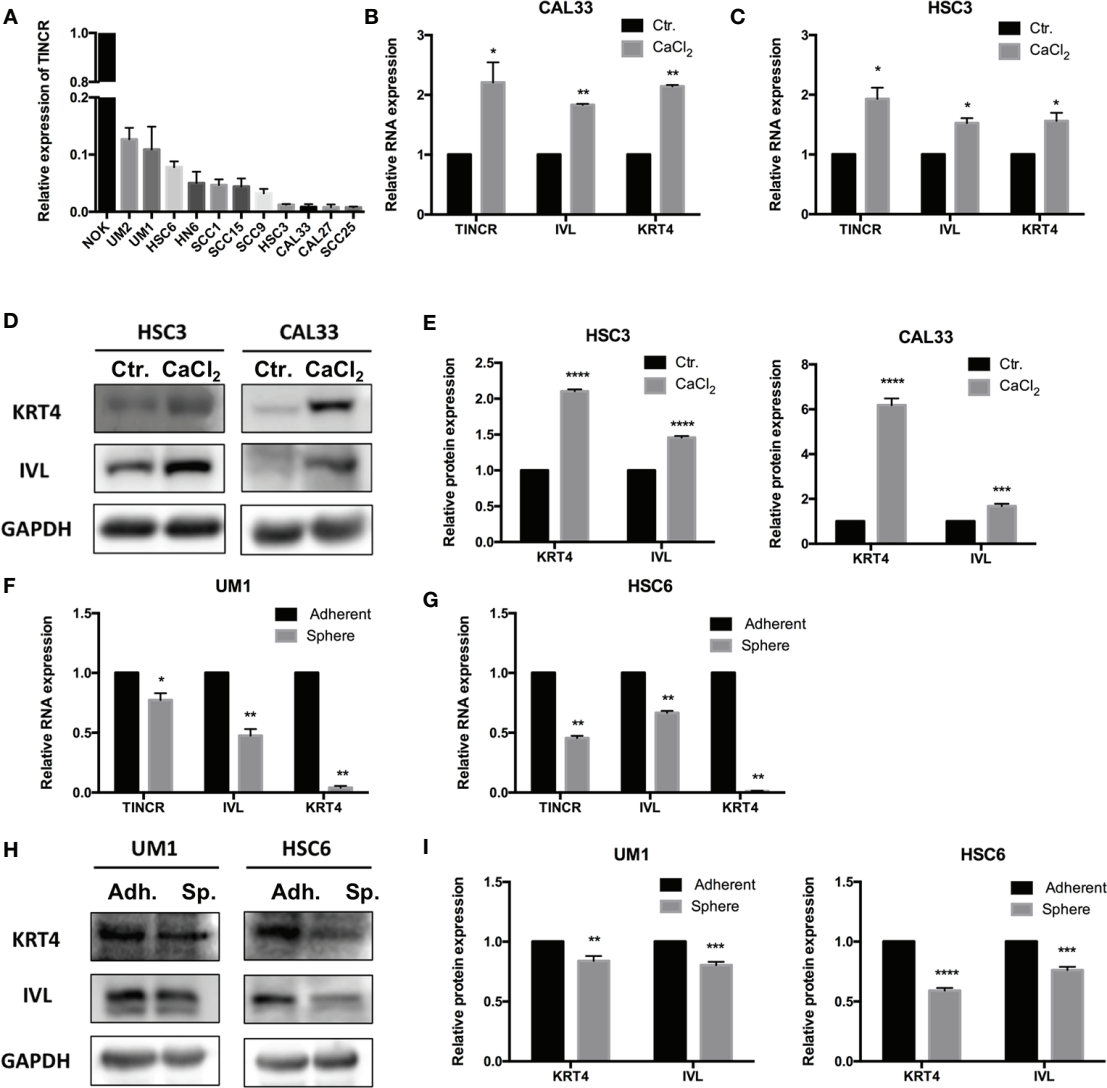
TINCR was involved in the cell differentiation of OSCC. **(A)** The expression of TINCR in OSCC cell lines and NOK cells was examined with qRT-PCR. The expression of TINCR in 11 OSCC cell lines was lower than that in NOK cells. **(B, C)** The expression of TINCR, IVL and KRT4 in OSCC cell lines was examined by qRT-PCR after cells were treated with CaCl_2_ (2.4 mmol/liter). **(D, E)** Western blot analysis showed that the protein expression of both IVL and KRT4 was upregulated after treatment with CaCl_2_. Quantification of the relative protein expression levels is shown. **(F, G)** qRT-PCR analysis revealed that the expression of TINCR, IVL and KRT4 was reduced in tumor spheres compared to adherent cells. **(H, I)** Western blot analysis showed that the protein expression of IVL and KRT4 was decreased in tumor spheres. Quantification of the relative protein expression levels is shown. *P < 0.05, **P < 0.01, ***P < 0.001, and ****P < 0.0001.

To choose appropriate cell lines to manipulate the expression of TINCR and examine its potential biological function, the expression of TINCR in several OSCC cell lines and normal oral keratinocytes (NOKs) was detected by using qRT-PCR. The results in **Figure 3A** showed that TINCR expression was markedly lower in a panel of 11 OSCC cell lines than that in NOK cells, which was consistent with the findings in the OSCC tissues. Notably, the expression of TINCR in UM1 and HSC6 cell lines was higher than that in CAL33 and HSC6 cells, which were then subjected to further experiments *in vitro*. To determine the subcellular localization of TINCR, we detected the expression level of TINCR in the cytoplasmic and nuclear fractions of HSC3 and HSC6 cells. As shown in **Supplementary Figures 2A, B**, qRT-PCR analysis revealed that 86.65 and 73.61% of the TINCR transcripts were detected in the nuclear fraction, respectively, while 13.35 and 26.39% were found in the cytoplasmic fraction. To examine alterations in TINCR expression during OSCC cell differentiation, we treated the OSCC cell lines HSC3 and CAL33 with CaCl_2_ (2.4 mmol/liter) for 2 days, and then total RNA and proteins were extracted. We found that the expression of TINCR was increased during OSCC cells calcium-induced differentiation. Meanwhile, both involucrin (IVL) and keratin 4 (KRT4), two commonly recognized differentiation-associated genes (15), were upregulated as well (**Figures 3B, C**). A Western blotting assay confirmed the increased levels of IVL and KRT4 proteins (**Figures 3D, E**). To further validate these findings, we treated the HSC3 cell line with all-trans retinoic acid (ATRA, 10 mmol/liter), another well-accepted differentiation inducer. As expected, ATRA increased expression levels of TINCR, IVL and KRT4 over time (**Supplementary Figures 3A, B**). Tumor sphere-forming assays were also performed to confirm our findings. Two OSCC cell lines, UM1 and HSC6, were cultured in non-adherent serum-free conditions, and total RNA and protein were collected for further analysis. As illustrated in **Figures 3F, G**, the mRNA levels of IVL and KRT4 were suppressed, accompanied by the down-regulation of TINCR when cell dedifferentiation occurred in tumor spheres. Similar results were also observed in the results of a Western blotting assay (**Figures 3H, I**). Correlation analysis showed that the expression of endogenous TINCR was positively correlated with expression levels of KRT4 and IVL (**Supplementary Figures 4A, B**). Moreover, a positive correlation between the basic expression level of TINCR and IVL or KRT4 was also observed in HNSCC patients from TCGA database using GEPIA (**Supplementary Figures 4C, D**). Taken together, our data suggested that TINCR may play a pivotal role in OSCC cell differentiation.

### TINCR Induced Cell Differentiation and Suppressed Migration, Invasion and Proliferation in OSCC Cells

**Figure 4:**
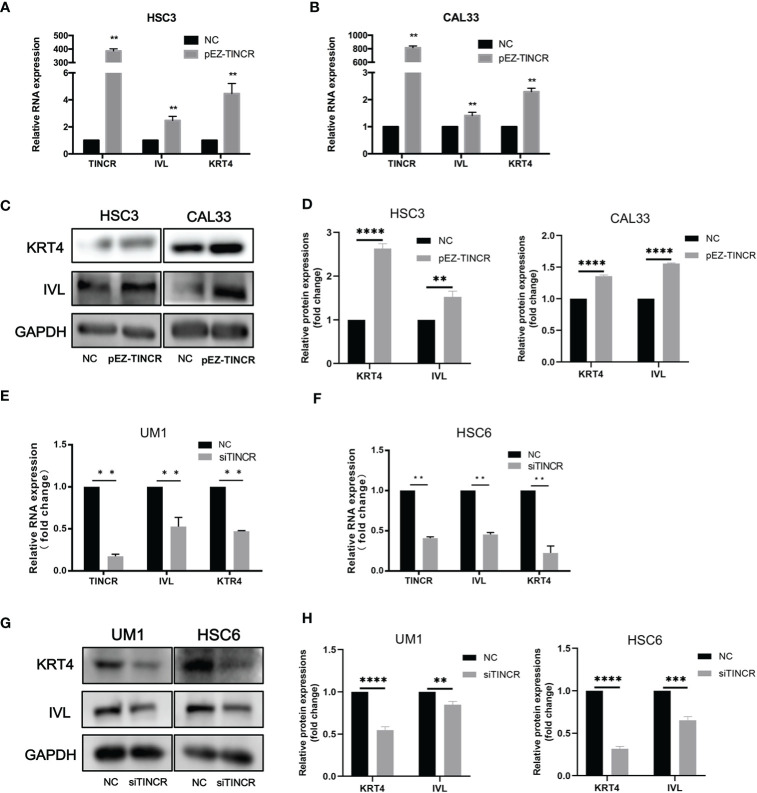
TINCR induced cell differentiation in OSCC cells. **(A, B)** The expression of TINCR, IVL and KRT4 in OSCC cell lines was measured by qRT-PCR after cells were transfected with the TINCR overexpression plasmid. **(C, D)** The protein expression of KRT4 and IVL was detected by Western blotting. Quantification of the relative protein expression levels is shown. **(E, F)** The expression of TINCR, IVL and KRT4 in OSCC cell lines was measured by qRT-PCR after cells were transfected with TINCR siRNA. **(G, H)** Western blot results showing the protein expression of IVL and KRT4. Quantification of the relative protein expression levels is shown. **P < 0.01, ***P < 0.001, and ****P < 0.0001.

**Figure 5:**
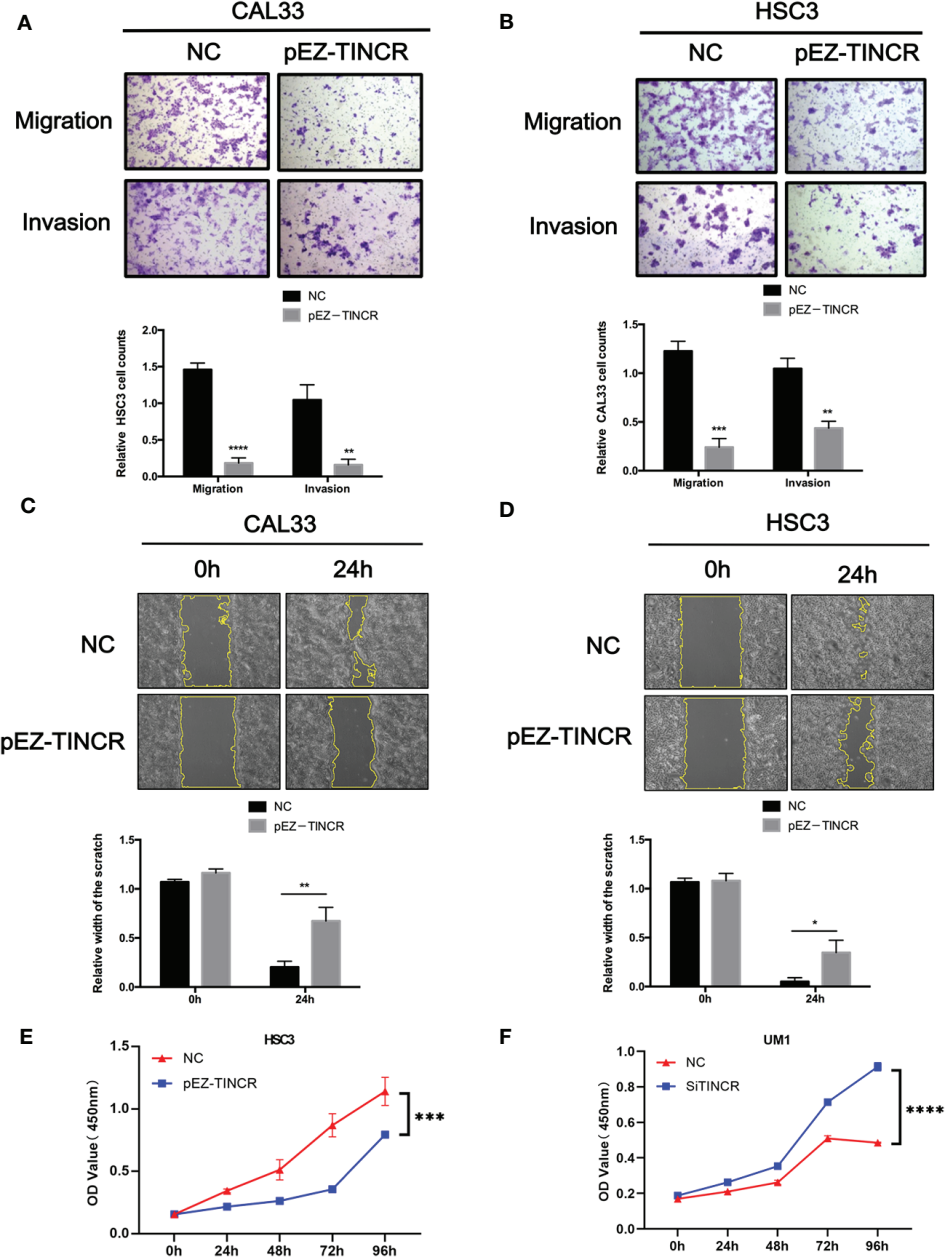
TINCR suppressed the migration, invasion, and proliferation of OSCC cells. **(A, B)** Transwell assays were used to evaluate the effect of TINCR overexpression on cell migration or invasion (upper panel). The relative numbers of cells that migrated or invaded are shown (lower panel). **(C, D)** Cell motility was evaluated by a wound healing assay in OSCC cells treated with the TINCR overexpression vector and control vector (upper panel). The relative width of the scratch in 0 h and 24 h is shown (lower panel). **(E)** Cell growth was evaluated by a CCK-8 assay in OSCC cells treated with the TINCR overexpression vector and control vector. **(F)** Cell growth was evaluated by a CCK-8 assay in OSCC cells treated with the TINCR siRNA and negative control. *P < 0.05, **P < 0.01, ***P < 0.001, and ****P < 0.0001.

To investigate the effect of TINCR on OSCC cell differentiation, functional analyses were performed by using the lentivirus-mediated stable overexpression of TINCR (pEZ-TINCR) in OSCC cell lines, including HSC3 and CAL33, both of which have low TINCR expression levels. The TINCR transfection efficiency was determined by qRT-PCR (**Figures 4A, B**). According to the qRT-PCR results, the elevated expression of TINCR led to the increased expression of differentiation-associated genes, including IVL and KRT4 (**Figures 4A, B**). The results of a Western blotting assay also demonstrated that protein levels of IVL and KRT4 were upregulated by TINCR overexpression (**Figures 4C, D**). In addition, overexpression of TINCR induced a dramatic change in cell-cell contact. Distinct spaces between cells became much less apparent (**Supplementary Figure 5**). Then, siRNA method was adopted to knock down TINCR in UM1 and HSC6 cell lines, in which TINCR was relatively highly expressed. As expected, TINCR depletion significantly reduced the expression of IVL and KRT4 at both the mRNA and protein levels relative to the negative control (**Figures 4E–H**). To study the effect of TINCR on cell migration and invasion, a Transwell assay was performed with TINCR-overexpressing cells. As shown in **Figure 5A**, overexpression of TINCR decreased the migration of CAL33 cells. Similar results were also observed in the HSC3 cell line (**Figure 5B**). Wound healing assays further confirmed that the exogenous upregulation of TINCR contributed to the repression of cell motility in CAL33 and HSC cells (**Figures 5C, D**). Moreover, to explore the effect of TINCR on tumor growth in *vitro*, a CCK-8 assay was performed. As shown in **Figure 5E, F**, overexpression of TINCR decreased the proliferation of HSC3 cells, while deletion of TINCR promoted cell growth in UM1 cells. These results were consistent with our findings in OSCC patients described above and supported the hypothesis that TINCR induces cell differentiation and functions as a tumor suppressor in OSCC.

### Overexpression of TINCR Inhibited the Tumorigenesis of OSCC *In Vivo*

**Figure 6:**
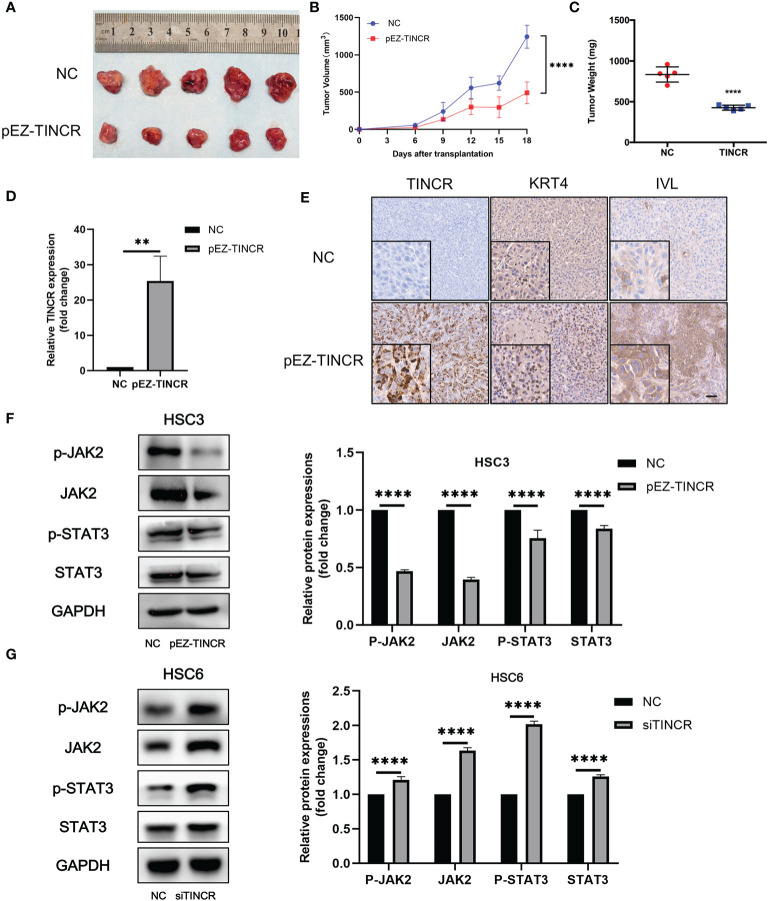
TINCR overexpression inhibited OSCC xenograft tumor growth. **(A)** Representative image of tumor xenografts after the subcutaneous injection of OSCC cells overexpressing TINCR or control cells. **(B)** Growth curves of the tumor volumes in the TINCR overexpression group and control group are shown. The tumor volumes were recorded every 3 days post-injection. **(C)** The tumor weight of the TINCR overexpression group was significantly lower than that of the control group. **(D)** qRT-PCR analysis verified that the expression of TINCR in the TINCR overexpression group was much higher than that in the control group. **(E)** RNAScope and IHC assays were used to detect the expression of TINCR, KRT4 and IVL in tumor xenografts, respectively. **(F)** Western blot analysis showed that the protein expression of p-JAK2, JAK2, p-STAT3 and STAT3 was remarkably inhibited in HSC3 cells after transfection with the TINCR overexpression plasmid (left panel). Quantitative analysis of p-JAK2, JAK2, p-STAT3 and STAT3 is shown (right panel). **(G)** Western blot analysis showed that the protein expression of p-JAK2, JAK2, p-STAT3 and STAT3 was significantly increased in HSC6 cells after transfection with TINCR siRNA (left panel). Quantitative analysis of p-JAK2, JAK2, p-STAT3 and STAT3 is shown (right panel). **P < 0.01, ****P < 0.0001.

To further validate the suppressive effects of TINCR on OSCC tumorigenesis *in vivo*, a xenograft model was established. The TINCR-overexpressing cells (pEZ-TINCR) and corresponding control cells (NC) were subcutaneously injected into BALB/c nude mice. As shown in **Figures 6A–C**, mice bearing cells overexpressing TINCR had smaller tumors with a lower tumor volume and weight than mice in the control group. Meanwhile, the tumor growth rate in the TINCR overexpression group was slower than that in the control group (**Figure 6B**). Furthermore, the expression level of TINCR in xenograft tumors was also confirmed by qRT-PCR and in situ hybridization assays (**Figures 6D, E**). Xenograft sections stained with IHC showed that the expression of IVL and KRT4 was increased in the TINCR overexpression group compared to the control group, supporting that TINCR may suppress tumor progression by inducing cell differentiation. Collectively, these data suggested that TINCR inhibits OSCC tumorigenesis *in vivo*.

### JAK2/STAT3 Signaling Pathway Was Involved in TINCR-Mediated Cell Differentiation

A couple of studies have proven that JAK2/STAT3 signaling plays a key role in modulating keratinocyte differentiation and epithelial carcinogenesis (16–19). Our previous data also suggested that JAK2/STAT3 signaling was involved in the tumorigenesis of HNSCC (20). To investigate the role of JAK2/STAT3 signaling in the TINCR-mediated cell differentiation of OSCC, the expression of key factors in JAK2/STAT3 signaling was detected after TINCR overexpression. Our results showed that TINCR overexpression significantly reduced the expression of JAK2, p-JAK2, STAT3, and p-STAT3 in HSC3 cells (**Figure 6F**). In contrast, all of these proteins were increased after TINCR deletion (**Figure 6G**). Taken together, our results suggested that JAK2/STAT3 signaling might be involved in TINCR-mediated cell differentiation.

## Discussion

Increasing evidence suggests that some lncRNAs act as oncogenes or tumor suppressors in tumorigenesis and progression. However, the molecular mechanisms have yet to be fully elucidated. In the present study, WGCNA was performed to explore the specific lncRNAs correlated with the pathogenesis of OSCC. A total of 23 gene modules were identified based on the microarray data, of which the blue module was significantly associated with OSCC and the lymph node status. A differentiation-related lncRNA, TINCR, was finally screened from the blue module by using GO enrichment and gene differential expression analysis. We verified that TINCR was dramatically downregulated in OSCC tissues, and was associated with pathological differentiation, lymph node metastasis, and poor survival in OSCC patients. We further showed that the expression of TINCR increased during cell differentiation and reduced during cell dedifferentiation. Functional analyses demonstrated that overexpression of TINCR mediated cell differentiation and suppressed the migration of OSCC cells. Furthermore, we also found that overexpression of TINCR decreased xenograft tumor volume and weight and promoted cell differentiation *in vivo*. Interestingly, down-regulation of TINCR led to the activation of JAK2/STAT3 signaling. These findings suggested that TINCR may serve as a tumor suppressor and induce cell differentiation by modulating JAK2/STAT3 signaling in OSCC.

LncRNAs have been widely reported to be involved in the proliferation, differentiation, apoptosis, invasion and metastasis of various human cancers (21–23). For example, the lncRNA LOWEG is remarkably downregulated in gastric cancer and suppresses migration and invasion in gastric cancer by acting as a tumor suppressor (24). In melanoma, ILF3-AS1 promotes cell proliferation, invasion and migration by negatively regulating miR-200b/a/429 (25). In colorectal cancer, GAPLINC is significantly upregulated and promotes cell migration and invasion by regulating miR-34a/c-MET (26). In addition, increased levels of H19 promote the invasion, angiogenesis, and stemness of glioblastoma cells. Upregulation of H19 in CD133^+^ glioblastoma cells is significantly related to the increased neurosphere formation of glioblastoma cells (27). All of these studies demonstrated that dysregulated lncRNAs are critical contributors to tumorigenesis and cancer progression. Moreover, accumulating evidence has revealed that lncRNAs exert crucial influences on the development of HNSCC, including the OSCC (28–30). H19, which is upregulated in OSCC, promotes cell proliferation and invasion (31). Huang et al. found that the down-regulation of NEAT1 represses cell proliferation and invasion in OSCC (32). Unfortunately, it remains a major challenge to effectively screen the specific lncRNAs from numerous lncRNA transcripts in high-throughput data. Few studies have focused on the clinical characteristics specific lncRNAs. WGCNA can transform gene expression data into co-expression modules and build correlation with clinical characteristics (33), which makes it a powerful tool for identifying modules of highly associated genes that can be widely applied to identify candidate biomarkers or therapeutic targets. In this study, a total of 23 gene modules were identified based on the WGCNA, of which the blue module was significantly related to the tissue and lymph node status. TINCR was then screened by both GO enrichment and gene differential expression analysis. We further validated that TINCR expression was strongly decreased in OSCC tissues compared with the NATs.

TINCR is aberrantly expressed in many human malignancies and closely associated with cancer occurrence, progression, invasion and metastasis, and prognosis (8, 34, 35). TINCR is downregulated in prostate cancer, and the low expression of TINCR is associated with advanced clinical T stage, lymph node metastasis, distant metastasis and poor prognosis (36). Yu et al. reported that upregulation of TINCR is negatively correlated with the overall survival of patients with colorectal cancer (37). TINCR is overexpressed in hepatocellular carcinoma and is related to tumor size, pathological differentiation, TNM classification, hematogenous metastasis and poor prognosis (34). However, its role in OSCC is still largely unknown. Here, the clinicopathological analysis was performed that revealed a close relationship between TINCR expression and pathological differentiation and lymph node metastasis in patients with OSCC. Lower TINCR expression is significantly associated with short survival. All of these results suggested that TINCR may serve as a tumor suppressor in OSCC progression.

TINCR plays a key regulatory role during human epidermal differentiation by interacting with a series of differentiation-related genes. A series of functional assays had confirmed that TINCR is necessary for epithelial cell differentiation and that its aberrant expression contributes to tumor occurrence and progression. An increasing number of studies have verified that TINCR serves as a regulatory factor in human squamous cell carcinoma, including breast cancer (38, 39), gastric cancer (10, 40), prostate cancer (36), lung cancer (41), hepatocellular carcinoma (34), bladder cancer (8), colorectal cancer (35), etc. In our study, we demonstrated that the expression of TINCR in OSCC cells was significantly upregulated, accompanied by an increase in IVL and KRT4 during cell differentiation induced by CaCl_2_. In contrast, the expression level of TINCR was dramatically suppressed, accompanied by IVL and KRT4 down-regulation, when OSCC cells were dedifferentiated in tumor spheres.

IVL is an important keratinocyte differentiation marker (42), and barrier protein (43). Chen et al. found that S100A has a prominent effect on IVL and therefore regulates esophageal cancer cell differentiation (44). KRT4 is a member of the keratin family, whose aberrant expression usually causes many diseases, and even cancer (45). According to sequence homology, keratins are classified into type I and type II clusters (46). Zhang et al. found that KRT4 is aberrantly expressed in human white sponge nevus (WSN) (47). Ohkura et al. observed the down-regulation of KRT4 in OSCC cells, but its upregulation in leukoplakia, suggesting that the expression level of KRT4 is closely related to malignancy in OSCC (48). Here, we explored the association among TINCR, KRT4, and IVL. Correlation analysis showed that the expression of endogenous TINCR was positively correlated with the expression levels of KRT4 and IVL. Moreover, the expression levels of IVL and KRT4 were remarkably decreased when TINCR was deleted. Conversely, the expression of IVL and KRT4 was distinctly increased in cells overexpressing TINCR. These results suggested that TINCR plays an important role in the process of epithelial differentiation and carcinogenesis of OSCC.

The Janus kinase (JAK)/signal transducer and activator of transcription (STAT) signaling pathway is an evolutionarily conserved pathway (49). It is activated in response to many protein ligands, such as cytokines and growth factors, which regulate various cellular processes, including cell proliferation, differentiation, and apoptosis (50, 51). JAK/STAT3 has been observed in numerous tumors and underpins a majority of features of cancer (52), including cell proliferation (53), antiapoptosis (54), angiogenesis (55), metastasis (56), and cancer stem cell maintenance (57). In addition, increasing evidence has proven that JAK/STAT3 signaling plays a key role in modulating keratinocyte differentiation and epithelial carcinogenesis (16–19). Amano et al. demonstrated that suppressing STAT3 activation by JAK inhibitors increases the levels of terminal differentiation and improves skin barrier function (17). Chan et al. also found that abrogation of STAT3 function leads to the significant repression of the growth of initiated keratinocytes and papilloma cells (18). In the present study, TINCR overexpression significantly suppressed the activation of JAK2/STAT3 signaling in OSCC cells, while the deletion of TINCR enhanced the JAK2/STAT3 signaling. Our findings indicated that JAK2/STAT3 signaling might be involved in the TINCR-mediated cell differentiation of OSCC. However, the underlying mechanism still needs further exploration.

In summary, our data suggest that TINCR functions as a tumor suppressor by inducing cell differentiation by modulating JAK2/STAT3 signaling in OSCC. TINCR may act as a prognostic biomarker and therapeutic target for OSCC.

## Data Availability Statement

The microarray data discussed in this study have been deposited in the NCBI Gene Expression Omnibus with the accession number GSE160042. The publicly available datasets analyzed in this study can be found in The Cancer Genome Atlas (TCGA) database (https://portal.gdc.cancer.gov)

## Ethics Statement

The studies involving human participants were reviewed and approved by Ethical Committee of Hospital of Stomatology, Sun Yat-sen University. The patients/participants provided their written informed consent to participate in this study. The animal study was reviewed and approved by Sun Yat-sen University Institutional Animal Care and Use Committee.

## Author Contributions

XL and JSH conceived the study and designed the experiments. ZZ, JH, CW, WW, and PY performed the *in vitro* and *in vivo* experiments and analyzed the data. JH and HL collected the patient samples and performed the clinical data analysis. HY and ZZ performed the bioinformatics analysis. ZZ, JH, and XL wrote the manuscript. All authors contributed to the article and approved the submitted version.

## Funding

This work was supported by the National Natural Science Foundation of China (81802704, 81772889, 81874128, 81702699, and 81572661).

## Conflict of Interest

The authors declare that the research was conducted in the absence of any commercial or financial relationships that could be construed as a potential conflict of interest.
