# Supplementary material for: Down-Regulation of Long Non-Coding RNA TINCR Induces Cell Dedifferentiation and Predicts Progression in Oral Squamous Cell Carcinoma
Source: Front Oncol. 2021 Feb 25;10:624752. doi: 10.3389/fonc.2020.624752 (PMC7959775; doi:10.3389/fonc.2020.624752)
Supplement: Supplementary file 1 [file DataSheet_1.pdf]

Supplementary Material

Supplementary Figures

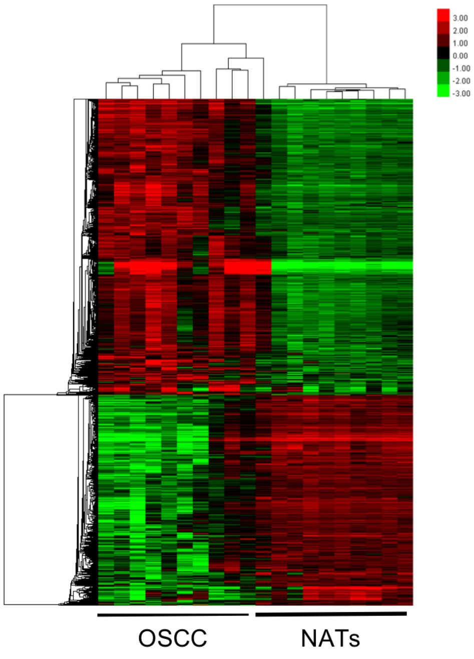

Supplementary Figure 1. The heatmap of mRNA and lncRNA expression profile of microarray. Base on the threshold of $P < 0.01$, and FC (fold change) ≥2, a total of 2592 differentially expressed genes (DEGs) in OSCC samples were screened when compared with non-cancerous adjacent tissues (NATs).

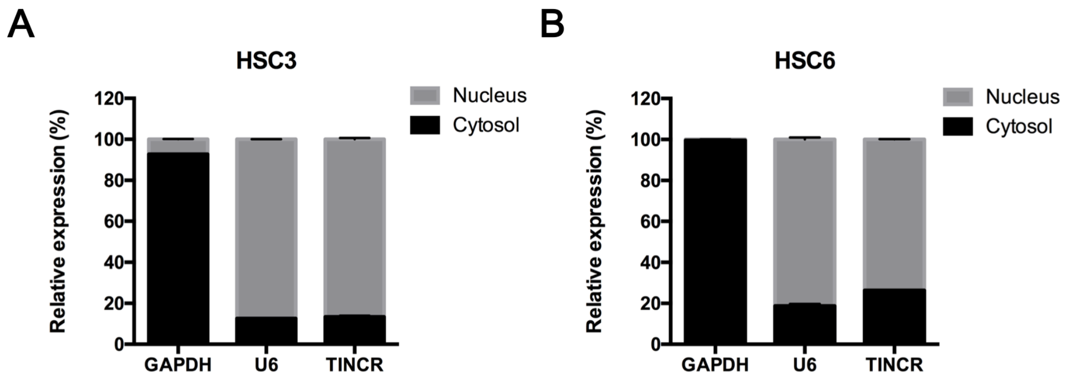

Supplementary Figure 2. The expression level of TINCR in nucleus or cytoplasm. The expression of TINCR in the nuclear and the cytoplasmic fraction in HSC3 **(A)** and HSC6 **(B)** was measured by qRT-PCR. U6 was used as a nuclear marker, and GAPDH was used as a cytosolic marker.

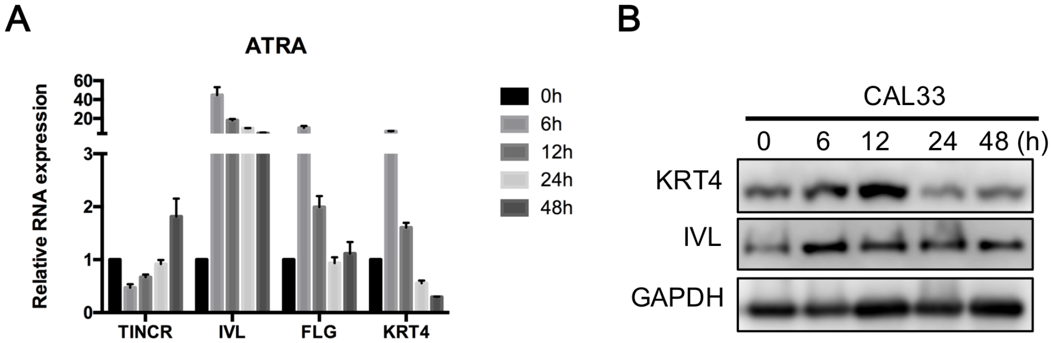

Supplementary Figure 3. TINCR was involved in cell differentiation induced by ATRA. (A) qRT-PCR analysis showed an increase expression of TINCR, IVL, FLG and KRT4 after CAL33 cell treated with all-trans retinoic acid (ATRA, 10mmol/L) at different periods of time. **(B)** Western blot analysis was performed to detected the protein expression of IVL and KRT4 in CAL33 cell treated with ATRA (10mmol/L) at different periods of time.

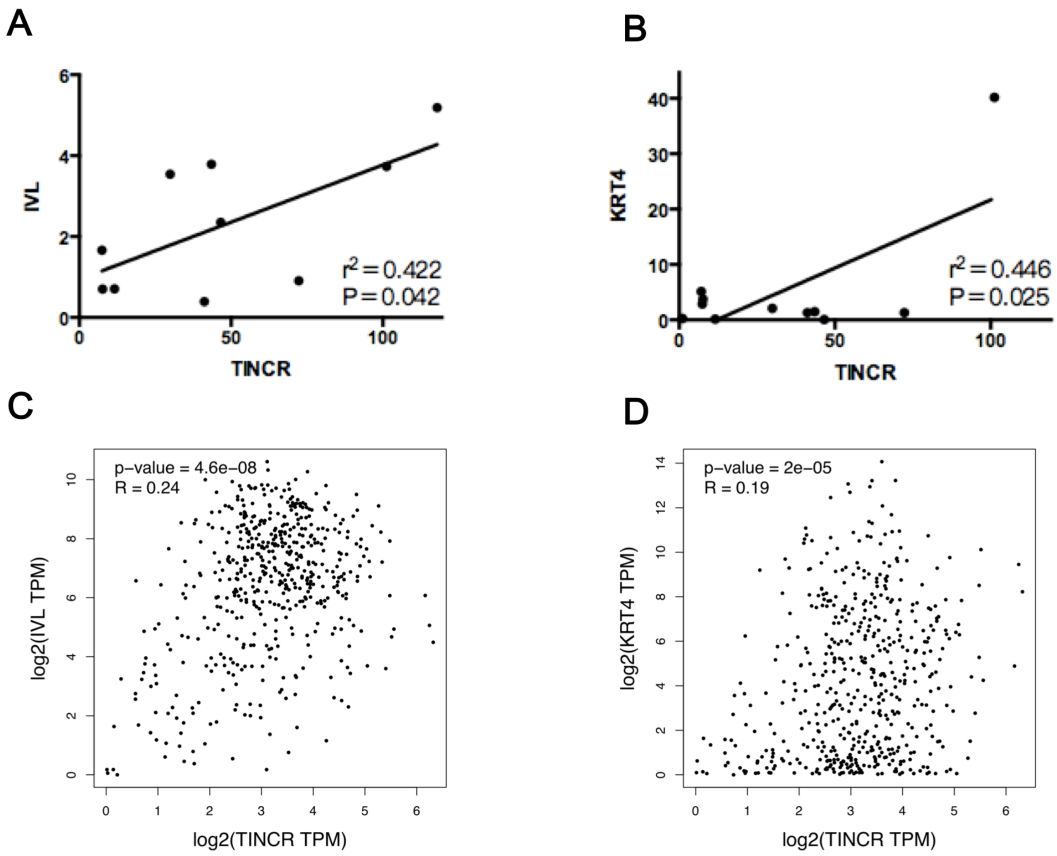

Supplementary Figure 4. The correlation analysis between the expression of TINCR and IVL and KRT4. The expression of endogenous TINCR was positively correlated with the expression levels of IVL **(A)** and KRT4 **(B)** in UM1 cell line. The expression level of TINCR was positively correlated with the expression levels of IVL **(C)** and KRT4 **(D)** in HNSCC patients from TCGA database.

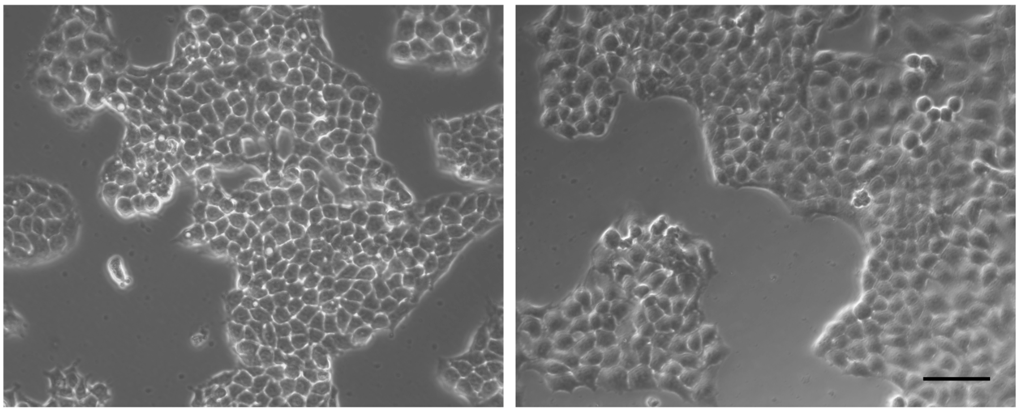

Supplementary Figure 5. Morphologic changement in cell differentiation mediated by TINCR overexpression. TINCR overexpression cells and corresponding control cells were cultivated in conventional medium and were observed under phase-contrast microscope.

Supplementary Table

Supplementary Table 1: List of primers of qRT-PCR.

Genes	Sequences
TINCR	Forward: TGTGGCCCAAACTCAGGGATACAT
	Reverse: AGATGACAGTGGCTGGAGTTGTCA
IVL	Forward: TCTGCCTCAGCCTTACTGTG
	Reverse: ACTGAGGGCAGGGGAGAG
KRT4	Forward: ACGTGGAGATTGACCCTGAG
	Reverse: TCGATGAAGGAGGCAAACTT
FLG	Forward: CTGGACACTCAGGTTCCCAT
	Reverse: TTTCGTGTTTGTCTGCTTGC
U6	Forward: CTCGCTTCGGCAGCACA
	Reverse: AACGCTTCACGAATTTGCGT
C5orf66-AS1	Forward: GAACGAACGAACGAAGCG
	Reverse: CCAGGTGGGGAGAAGTGG
GAPDH	Forward: CGCTGAGTACGTCGTGGAGTC
	Reverse: GCTGATGATCTTGAGGCTGTTGTC
